# Neural Oscillatory Mechanisms Underlying Step Accuracy: Integrating Microstate Segmentation with eLORETA-Independent Component Analysis

**DOI:** 10.3390/brainsci15040356

**Published:** 2025-03-29

**Authors:** Kohei Okuyama, Kota Maeda, Ryosuke Yamauchi, Daichi Harada, Takayuki Kodama

**Affiliations:** 1 Department of Physical Therapy, School of Health Sciences, Bukkyo University, Kyoto 604-8418, Japan; 2 Graduate School of Health Sciences, Kyoto Tachibana University, Kyoto 607-8175, Japan; onebase824@gmail.com (K.M.); h901522007@st.tachibana-u.ac.jp (R.Y.); kodama-t@tachibana-u.ac.jp (T.K.); 3 Department of Physical Therapy, Faculty of Health Sciences, Kyoto Tachibana University, Kyoto 607-8175, Japan; a903021139@st.tachibana-u.ac.jp

**Keywords:** anterior cingulate cortex, mobile brain/body imaging, EEG, eLORETA-ICA, error monitoring, microstate segmentation analysis, motor control, neural oscillatory mechanisms, step accuracy

## Abstract

Background/Objectives: Precise stepping control is fundamental to human mobility, and impairments increase fall risk in older adults and individuals with neurological conditions. This study investigated the cortical networks underlying stepping accuracy using mobile brain/body imaging with electroencephalography (EEG)-based exact low-resolution electromagnetic tomography-independent component analysis (eLORETA-ICA) and microstate segmentation analysis (MSA). Methods: Sixteen healthy male participants performed a precision stepping task while wearing a mobile EEG system. Step performance was quantified using error distance, measuring deviation between target and heel contact points. Preprocessed EEG data were analyzed using eLORETA-ICA and MSA, with participants categorized into high- and low-performing groups. Results: Seven microstate clusters were identified, with the anterior cingulate cortex (ACC) showing the highest microstate probability (21.15%). The high-performing group exhibited amplified theta-band activity in the ACC, enhanced activity in the precuneus and postcentral gyrus, and suppressed mu- and beta-band activity in the paracentral lobules. Conclusions: Stepping accuracy relies on a distributed neural network, with the ACC playing a central role in performance monitoring. We propose an integrated framework comprising the following systems: error monitoring (ACC), sensorimotor integration (paracentral lobules), and visuospatial processing (precuneus and occipital regions). These findings highlight the importance of neural oscillatory mechanisms in precise motor control and offer insights for rehabilitation strategies and fall prevention programs.

## 1. Introduction

Stepping accuracy is crucial for maintaining balance and mobility, particularly during gait initiation, as it determines how effectively an individual can transition from a stationary position to walking [1]. Deficits in this initial step may compromise stability, leading to an increased risk of falls, particularly among older adults [2,3] and individuals with neurological disorders such as Parkinson’s disease [4,5]. The initial step movement plays a crucial role in maintaining balance and adapting to environmental changes. A better understanding of the cortical mechanisms underlying step accuracy could provide insights into fall prevention strategies and rehabilitation interventions for at-risk populations, including individuals with Parkinson’s disease and older adults.

In this context, stepping performance refers to the ability to place the foot accurately in space and time during gait [6,7,8], requiring precise motor coordination and cognitive control [9]. This process engages multiple brain regions, including the supplementary motor area, primary motor cortex, prefrontal cortex, primary somatosensory cortex, posterior parietal cortex (PPC), and anterior cingulate cortex (ACC) [10]. However, the specific neural mechanisms underlying step accuracy remain unclear.

Traditional assessments of gait and stepping performance rely on biomechanical methods, including force plates [11,12], pressure-sensitive mats [13], motion capture systems [14], and inertial measurement units [15]. While these methods provide valuable kinematic and kinetic data, they offer limited insight into the neural mechanisms governing step accuracy. Functional neuroimaging techniques, such as functional magnetic resonance imaging (fMRI), have enhanced our understanding of motor-related brain function but are restricted to simulated movement paradigms in supine positions, limiting their ecological validity for studying real-world locomotion [10]. Functional near-infrared spectroscopy provides greater mobility but lacks the temporal resolution required to analyze rapid neural dynamics during step initiation [16].

To overcome these limitations, mobile brain/body imaging (MoBI) has emerged as a promising approach. MoBI integrates mobile electroencephalography (EEG) with motion capture and physiological monitoring, enabling real-time investigation of brain dynamics during natural movement [17,18]. Despite early challenges such as motion artifacts and non-neural signal contamination, advancements in signal processing have significantly improved artifact removal [19,20], enabling MoBI to be applied to dynamic tasks, including juggling [21] and skateboarding [22]. These improvements have expanded the application of MoBI to the study of step accuracy, allowing for the real-time analysis of neural activity at the precise moment of step execution. In contrast to traditional methods, MoBI provides both high temporal resolution and ecological validity, making it uniquely suited to studying the neural correlates of step accuracy in natural walking conditions.

Previous studies have identified neural signatures associated with gait control. Increased theta-band activity in the ACC has been linked to error monitoring and balance adjustments [23,24], while reductions in mu- and beta-band activity in the sensorimotor cortex and PPC reflect motor planning and execution [25,26]. Additionally, delta-band fluctuations in motor-related regions have been associated with gait control and feedback processing [27,28]. However, most of these studies have focused on steady-state walking rather than the initial step movement, which is critical for understanding gait initiation and step accuracy. While previous MoBI studies have investigated cortical activity during continuous walking, little is known about the precise neural mechanisms underlying the initiation of stepping, particularly in relation to step accuracy. In contrast to prior studies that primarily focused on gross locomotor patterns, our study examined the fine-grained neural dynamics of step execution.

To address this gap, this study employed exact low-resolution electromagnetic tomography–independent component analysis (eLORETA-ICA) to examine cortical activity associated with step accuracy. eLORETA-ICA combines source localization with independent component analysis, enabling the decomposition of EEG signals across multiple frequency bands with high spatial resolution [29,30,31]. Additionally, microstate segmentation analysis (MSA) enables tracking of temporal network dynamics at millisecond precision, offering a detailed view of the cognitive and motor processes underlying stepping accuracy [32,33].

This study investigated how different cortical regions contribute to step accuracy during gait initiation. By analyzing EEG data using eLORETA-ICA and MSA, this research aimed to clarify the neural mechanisms underlying step accuracy and provide new insights into motor planning, execution, and error monitoring processes. Understanding these neural dynamics could help inform the development of targeted interventions for populations at risk of impaired stepping performance, including older adults and individuals with neurological disorders.

## 2. Materials and Methods

### 2.1. Participants

This study included 16 healthy male paid volunteers (mean age: 23.19 ± 2.66 years; mean height: 169.86 ± 4.51 cm). Participant characteristics are presented in Table 1. The exclusion criteria were female sex, individuals with dementia or suspected dementia (Mini-Mental State Examination [MMSE] score < 24), and those with orthopedic issues that could affect walking. The MMSE was used to exclude participants with potential cognitive impairments that could confound the assessment of stepping performance, which requires cognitive resources such as attention and executive function. This exclusion criterion was implemented to ensure that the observed neural activity primarily reflects motor control mechanisms in the absence of significant cognitive decline. Female participants were excluded owing to the known susceptibility of EEG activity to hormonal fluctuations, particularly those associated with the menstrual cycle, which can affect EEG stability and signal patterns [34,35]. Given the exploratory nature of this study, male participants with higher EEG stability were selected to minimize the potential variability in EEG measurements. Furthermore, the participants were required to abstain from caffeine, alcohol, and tobacco use for 24 h before the experiment and confirm that they were not currently taking any medications. They also reported their sleep duration from the previous night, as sleep can affect EEG measurements [36]. The dominant foot was identified as the foot used to kick a ball [37].

### 2.2. Experimental Methodology

This study was conducted between December 2023 and March 2024 in the Exercise Physiology Function Assessment Laboratory at Kyoto Tachibana University, Kyoto, Japan.

#### 2.2.1. Stepping Task Protocol

**Figure 1:**
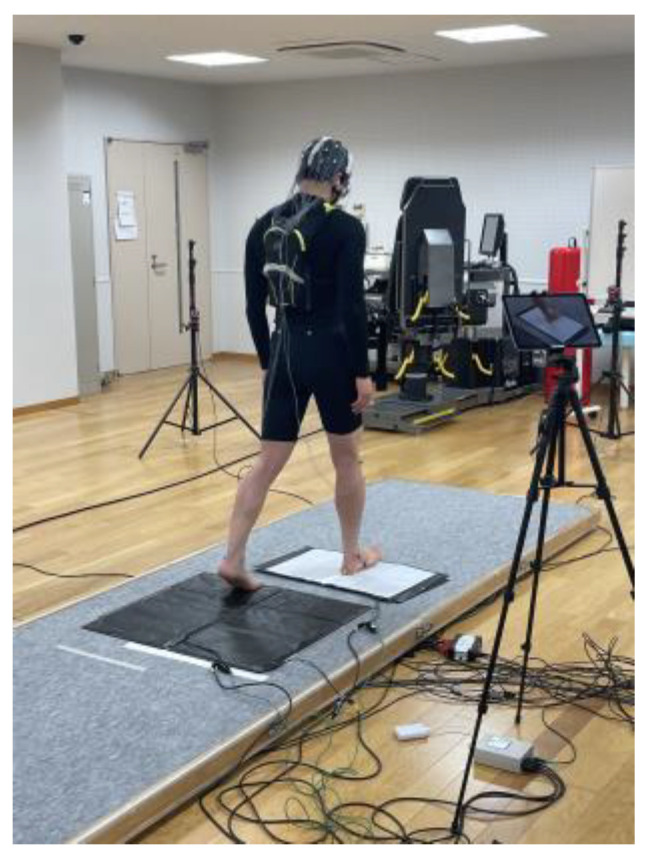
Experimental setup. The participant is wearing a 28-electrode EEG cap while standing on the pressure-sensitive mat system. The black mat in front of the participant is the initial position mat, and the white mat is the target position mat. Small markers were placed on the posterior and lateral sides of the calcaneus to precisely measure heel contact points. An iPad was used to record stepping movements. Participants performed 20 stepping trials, aiming to align their heels with the designated target point. EEG: electroencephalography.

Participants performed a precision stepping task while their brain activity, eye movements, and muscle activity were recorded. Figure 1 shows the experimental setup with a participant wearing the EEG cap and standing on the pressure-sensitive mat system.

**Figure 2:**
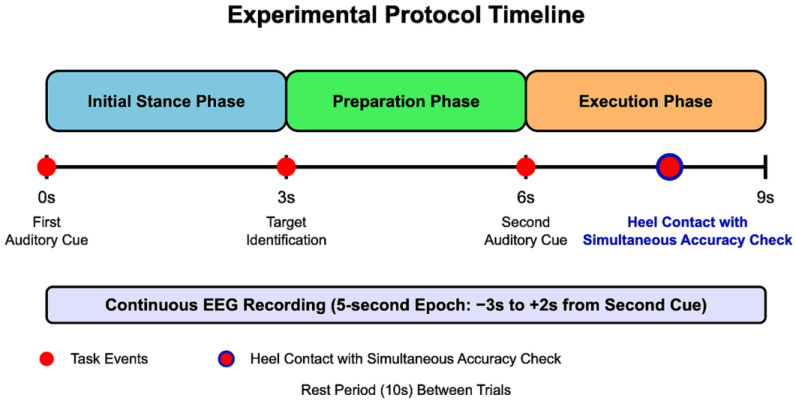
Experimental protocol timeline. The figure shows the temporal sequence of events during each stepping trial, including the initial stance, preparation, and execution phases. EEG recording epochs were extracted from 3 s before to 2 s after the second auditory cue.

For each trial, participants followed a standardized protocol as illustrated in Figure 2:

1. Initial Stance Phase (0–3 s): Participants stood in a stable, comfortable, and natural posture with their weight evenly distributed between both feet. An auditory cue signaled the beginning of the trial.
2. Preparation Phase (3–6 s): Participants visually identified the target point (marked with an 8 mm circular marker) and prepared their stepping. They were instructed to maintain their initial stance during this period.
3. Execution Phase (6–9 s): A second auditory cue prompted participants to take a single step forward with their dominant leg, aiming to align the calcaneal tuberosity with the designated target point. No specific instructions regarding step speed were given to ensure natural movement patterns. After heel contact, participants simultaneously performed a visual accuracy check by comparing their heel position to the target marker before returning to the starting position for the next trial.

A rest period of 10 s was provided between trials to prevent fatigue. Each participant completed 20 stepping trials with the same target location.

#### 2.2.2. Data Acquisition Systems

##### EEG Recording

EEG data were recorded using active electrodes and the Polymate V AP5148 biological signal acquisition device (Miyuki Giken Co., Ltd., Tokyo, Japan). Measurements were performed at 28 electrode sites according to the international 10–10 system [38,39], including Fpz, Fz, Cz, Pz, Oz, FP1, FP2, F7, F8, F3, F4, C3, C4, C5, C6, P3, P4, P7, P8, O1, O2, T7, T8, CPz, CP1, CP2, PO3, and PO4. The ground electrode was placed on the spinous process of the seventh cervical vertebra, and the reference electrode was placed on the right earlobe. Recordings were sampled at 1 kHz, and electrode impedance was maintained below 10 kΩ. To minimize artifacts, participants were instructed to limit head movements and eye blinks during the stepping task.

##### Supplementary Physiological Recordings

In addition to EEG recordings, the following physiological signals were recorded simultaneously:

1. Electrooculography (EOG): Vertical EOG was recorded by placing electrodes above and below the dominant eye to detect eye movement and blink artifacts.
2. Electromyography (EMG): Surface EMG was recorded bilaterally from the tibialis anterior muscles. Electrode placement followed SENIAM’s recommendations [40]. A bipolar derivation was used with an inter-electrode distance of 2 cm. The EMG signals were sampled at 1 kHz.

##### Pressure-Sensitive Mat System

For accurate detection of stepping timing, a pressure-sensitive mat system developed by Caretech Inc. (Japan) was employed. This system featured the following characteristics:

1. Sensor Configuration: Three pressure-sensitive mats were positioned as follows:
  - Two mats at the initial standing position (one for the right foot and one for the left foot);
  - One mat at the target position (around the heel contact area).
2. Data Acquisition and Synchronization: Signals from each mat sensor were directly synchronized with the EEG recording system. Trigger signals were collected at the same sampling rate as the EEG (1 kHz), with a temporal precision of ±5 ms.
3. Trigger Functions: The mat sensors generated trigger signals to accurately detect the following two key events:
  - Moment of unloading of the dominant foot (decrease in pressure on the initial position mat at step initiation);
  - Moment of heel contact (increase in pressure on the target position mat).

##### Video Recording System

Stepping movements were recorded using an iPad Air (Apple Inc., Cupertino, CA, USA; 11-inch, 4th generation; resolution: 1920 × 1080 pixels; frame rate: 30 frames per second). The device was positioned at a fixed height of 1.5 m and oriented as close to perpendicular to the plane of motion as possible to minimize perspective distortion.

#### 2.2.3. Step Performance Assessment

Step performance was quantified using the error distance (ED), defined as the spatial deviation between the target point and the actual heel contact point. To ensure accurate measurement, the following procedure was employed:

1. Video Processing: Videos of the stepping trials were processed using the Fiji software (Fiji Is Just ImageJ, Version 1.54f) [38] to extract individual frames corresponding to heel contact moments.
2. Calibration: A 10 mm reference marker was placed in each video frame for accurate spatial calibration and distance measurement.
3. Reference Points: The heel contact position was defined using the following anatomical landmarks:
  - X-axis reference point: Dorsal (posterior) side of the calcaneal tuberosity;
  - Y-axis reference point: Lateral side of the calcaneal tuberosity.
4. Measurement: These reference points were marked with small (8 mm diameter) high-contrast circular markers to enhance visibility. ED was calculated as the Euclidean distance between the target point (xt, yt) and the heel contact point (xh, yh) using the following formula.

$$d=\sqrt{{\left(X_{target}-X_{heel}\right)}^{2}+{\left(Y_{target}-Y_{heel}\right)}^{2}}$$

All measurements were recorded in millimeters. For each participant, the mean ED across all 20 trials was calculated and used for subsequent analysis.

### 2.3. Data Analysis

#### 2.3.1. EEG Preprocessing

EEG data preprocessing was performed using the multimodal EEG analysis program EMSE Suite (CORTECH SOLUTIONS, Inc., Wilmington, NC, USA; Miyuki Giken Co., Ltd., Japan). The preprocessing pipeline comprised the following steps:

1. Filtering: Initially, continuous EEG data were filtered using the following parameters:
  - High-pass filter: 1.5 Hz (to remove low-frequency drift);
  - Low-pass filter: 30 Hz (to remove high-frequency noise);
  - Zero-phase digital filtering was implemented using the filter pipeline in EMSE to minimize phase distortion.
2. Power Line Noise Removal: A band-stop filter (notch filter) was applied to selectively remove power line noise:
  - Since the experiment was conducted in western Japan (Kyoto), where the commercial power frequency is 60 Hz, a band-stop filter centered at 60 Hz with a bandwidth of 2 Hz was applied.
3. EOG Artifact Removal: Eye movement and blink artifacts were removed using the template-based EOG artifact removal function implemented in EMSE. Using the simultaneously recorded EOG signal as a reference, typical eye movement and blink patterns were defined as templates, and these patterns were detected and removed from each EEG channel.
4. Segmentation: The preprocessed continuous data were segmented according to each stepping trial as follows:
  - From 3 s before to 2 s after the presentation of the second auditory cue (stepping initiation instruction);
  - Each segment covered a 5 s time window, encompassing the preparation phase through post-movement completion.
5. Quality Control: The segmented data underwent quality verification through the following procedures:
  - Automatic rejection of epochs containing values exceeding ±100 μV;
  - Visual inspection by experienced researchers (K.O. and T.K.) to identify any remaining artifacts;
  - Re-referencing to average reference to minimize the influence of the reference electrode location.

#### 2.3.2. eLORETA-ICA Analysis

Source analysis was performed on the preprocessed EEG data using the eLORETA software (version 20140711, http://www.uzh.ch/keyinst/loreta.htm, accessed on 1 August 2023) [41]. The following analytical procedures were employed:

1. Source Estimation: Preprocessed scalp EEG data were transformed into current density distributions in the brain using the eLORETA algorithm. eLORETA features minimal localization bias for source estimation at 6239 cortical gray matter voxels with 5 mm spatial resolution within a realistic head model [42].
2. Frequency-Domain ICA Application: The frequency-domain ICA (transposed fICA networks) module integrated into the eLORETA was directly applied to the preprocessed EEG data. This approach decomposes cortical electrical activity across multiple frequency bands into independent components (ICs), enabling simultaneous analysis of multiple frequency bands that would be difficult with conventional structural source analysis [29,31]. This module was configured to analyze the following four frequency bands:
  - Delta band (1.5–3.5 Hz);
  - Theta band (4–7.5 Hz);
  - Alpha/mu band (8–13 Hz, with the mu rhythm specifically referring to activity recorded over the sensorimotor areas) [43];
  - Beta band (13.5–30 Hz).

This approach enables the extraction of functionally independent brain network components by applying ICA in the source space after decomposing signals into frequency bands.

3. Component Evaluation: Three experienced researchers (K.O., K.M., and T.K.) evaluated the resulting components and collaboratively classified them into neural activity and artifact components based on their spatial and spectral characteristics.

#### 2.3.3. Microstate Segmentation Analysis

The identified ICs from eLORETA-ICA were further analyzed using the eLORETA clusters/microstates module integrated within the eLORETA software to assess patterns of neural activity during the stepping task. This analysis identifies quasi-stable patterns of brain activity (microstates) that represent distinct functional states. The procedure comprised the following steps:

1. Clustering Algorithm: The k-means clustering algorithm was applied to the source-space data to identify prototypical microstate maps.
2. Number of Clusters: Based on previous literature on gait-related brain activity [10], the number of clusters was set to seven, corresponding to brain regions previously identified as associated with gait and stepping motions. The optimal number of clusters was verified using the cross-validation criterion, which confirmed that seven clusters provided the best balance between model complexity and explanatory power.
3. Microstate Parameter: For each identified microstate, its probability (the likelihood of each microstate occurring during the task) was calculated and used for subsequent analysis.

#### 2.3.4. Statistical Analysis

After completing the EEG analysis using eLORETA-ICA, the participants were categorized into the following two groups based on their stepping performance:

1. High-Performing (HiP) Group: Participants with smaller ED values (below the median value), indicating more accurate stepping (n = 8).
2. Low-Performing (LoP) Group: Participants with larger ED values (above the median value), indicating less accurate stepping (n = 8).

The median split method was chosen based on its established use to robustly dichotomize continuous variables for group comparisons [44]. To ensure the robustness of our findings, we verified that the two groups significantly differed in their stepping performance using an independent samples *t*-test.

Statistical comparisons between the HiP and LoP groups were conducted using the statistics eLORETA SnPM 26 function (multiple paired *t*-tests with nonparametric randomization) included in the analysis program. This approach uses permutation tests to identify statistically significant differences between groups while controlling for multiple comparisons. The significance level was set at *p* < 0.05.

### 2.4. Ethical Considerations

Before the initiation of this study, all participants were provided with both written and verbal explanations of the purpose and content of the study through a research information sheet. All participants provided informed consent to participate in this study via signatures on a consent form. This study was conducted in accordance with the Declaration of Helsinki and was approved by the Research Ethics Committee of Kyoto Tachibana University (approval number: 23–44).

## 3. Results

### 3.1. Quality of EEG Data and Preprocessing Efficacy

**Figure 3:**
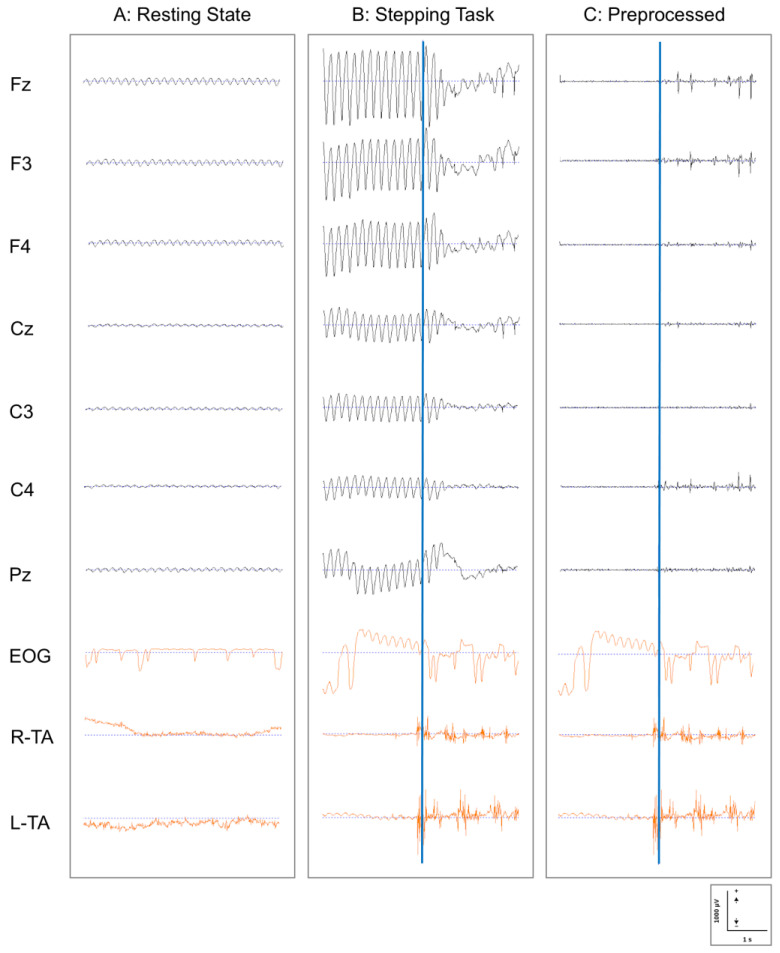
EEG, EOG, and EMG recordings during resting state and stepping task: before and after preprocessing. (**A**) Resting state: raw data during the resting condition; (**B**) stepping task: raw data during the stepping task showing significant movement artifacts; and (**C**) preprocessed: the same stepping task data after application of the artifact removal pipeline. Displayed channels include frontal (Fz, F3, and F4), central (Cz, C3, and C4), and parietal (Pz) EEG recordings (black); vertical EOG; and bilateral tibialis anterior EMG (R-TA and L-TA) (orange). Blue vertical lines in panels B and C indicate stepping trigger moments, marking the initiation of dominant foot movement. EEG signals are presented with conventional polarity (positive deflections upward). The preprocessing pipeline effectively removed movement artifacts while preserving relevant neural signals. Scale bars: 1000 μV (vertical) and 1 s (horizontal). EEG: electroencephalography; EMG: electromyography; EOG: electrooculography.

Figure 3 presents representative EEG, EOG, and EMG recordings from a participant during the following three conditions: resting state (A); stepping task before preprocessing (B); and stepping task after preprocessing (C). The comparison between the raw and preprocessed stepping data demonstrates the efficacy of our artifact removal methodology. Movement-related artifacts visible in panel B were effectively eliminated in panel C while preserving the neurophysiological signals of interest. These preprocessed data were subsequently used for eLORETA-ICA analysis to identify neural components associated with stepping performance.

### 3.2. IC and Microstate Analysis

Using eLORETA-ICA, 13 ICs were identified from EEG recordings obtained during the stepping task. Of these, 10 ICs (IC-1, IC-2, IC-3, IC-5, IC-7, IC-9, IC-10, IC-11, IC-12, and IC-13) were associated with neural network activity, while the remaining ICs (IC-4, IC-6, and IC-8) were classified as artifacts (see Appendix A for visualization).

**Figure 4:**
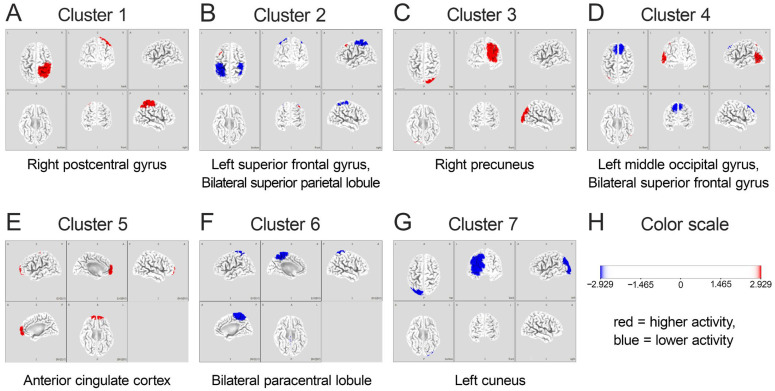
Seven clusters calculated using eLORETA microstates. Each panel (**A**–**G**) represents an independent microstate cluster: (**A**) Cluster 1: right postcentral gyrus; (**B**) Cluster 2: left superior frontal gyrus/bilateral superior parietal lobule; (**C**) Cluster 3: right precuneus; (**D**) Cluster 4: left middle occipital gyrus/bilateral superior frontal gyrus; (**E**) Cluster 5: anterior cingulate cortex; (**F**) Cluster 6: bilateral paracentral lobule; (**G**) Cluster 7: left cuneus. The color scale (**H**) represents t-values (t = 2.929), with red and blue indicating higher and lower activities, respectively. Brain images are displayed from various perspectives, with medial views chosen when either maximum or minimum activity is located in medial brain regions to optimally visualize both the activation and deactivation patterns of each cluster. eLORETA: exact low-resolution electromagnetic tomography.

The activity was analyzed across four frequency bands (theta, alpha, mu, and beta) for each of the 10 ICs, and eLORETA microstate analysis grouped them into seven clusters (Clusters 1–7). These seven microstate clusters are presented in Figure 4, with each cluster representing a distinct functional network. The detailed anatomical locations of these clusters are presented in Table 2.

The detailed frequency-band characteristics of each cluster are listed in Table 3. The microstate probability analysis revealed the relative contribution of each frequency band within the seven clusters. Notably, Cluster 5 (corresponding to the ACC) exhibited the highest overall microstate probability of 21.15%, with a particularly strong representation in the theta band (38.46%).

### 3.3. Comparison Between HiP and LoP Groups

Based on stepping performance quantified by ED, participants were categorized into HiP (n = 8; mean ED = 12.8 ± 2.6 mm) and LoP (n = 8; mean ED = 23.1 ± 4.1 mm) groups using a median-split method. The between-group difference in ED was statistically significant (t(14) = 5.02, *p* < 0.001), confirming the validity of this grouping approach.

When comparing neural activity patterns between the HiP and LoP groups, significant differences emerged across several brain regions. The HiP group demonstrated amplified theta-band activity in the ACC (Cluster 5, *p* < 0.05; Cohen’s d = 1.57), enhanced activity in the precuneus (Cluster 3) and right postcentral gyrus (Cluster 1), and significant attenuation of mu- and beta-band activity in the bilateral paracentral lobules (Cluster 6, *p* < 0.05).

## 4. Discussion

In this study, we analyzed brain activity during initial step movement using eLORETA-ICA and MSA, comparing HiP and LoP stepping groups. Our analysis identified seven microstate clusters, with Cluster 5 (ACC) exhibiting the highest probability of theta band activity. The HiP group demonstrated amplified ACC theta-band activity, increased activity in the precuneus and right parietal lobe (postcentral gyrus), and the suppression of mu- and beta-band activity in the bilateral paracentral lobules.

### 4.1. Amplified ACC Theta-Band Activity: Role in Performance Monitoring

The ACC is widely recognized for its involvement in error detection and conflict monitoring, playing a crucial role in adjusting motor control systems [45,46]. Particularly noteworthy is the association between theta band activity and cognitive control functions [47]. Our finding of enhanced theta-band activity in the ACC in the HiP group extends these observations to the domain of stepping accuracy, providing novel insights into the neural mechanisms of precise locomotion.

Accurate stepping demands the continuous integration of visual and somatosensory information while rapidly detecting and correcting errors. The amplified ACC theta activity in the HiP group indicates more efficient performance monitoring and top-down control, a critical neural signature differentiating high performers from their counterparts. This observation directly addresses our primary research question regarding the neural mechanisms underlying stepping accuracy, establishing the ACC as a central hub for error monitoring during precision stepping tasks.

These results align with multiple lines of evidence from locomotor research. Studies on brain–computer interface (BCI)-controlled treadmill walking have reported increased low-frequency activity in the ACC associated with error monitoring and motor learning networks [27]. Similarly, investigations using split-belt treadmill adaptation paradigms have demonstrated increased theta power in the ACC during early adaptation phases [23], providing converging evidence for the role of ACC theta oscillations in locomotor adaptation. During perturbations to standing balance, the ACC functions as a hub for theta-band coupling, suggesting its broader roles in various aspects of balance control [24].

Our findings reveal that individuals with superior stepping accuracy engage in enhanced ACC-mediated monitoring processes, which likely facilitate the detection of minor deviations between intended and actual foot placement. This real-time error detection system appears crucial for the precise adjustments necessary to achieve accurate stepping. The theta oscillations may serve as a communication mechanism between monitoring systems and motor execution areas, enabling rapid corrections to ensure accurate foot placement.

While traditional neuroimaging studies using fMRI have emphasized the role of the supplementary motor area and primary motor cortex in gait control [48], our electrophysiological approach reveals the dynamic temporal characteristics of ACC involvement. These findings suggest that precise stepping relies not merely on motor execution capabilities but critically depends on cognitive monitoring processes mediated by the ACC.

### 4.2. Suppression of Mu- and Beta-Band Activity in Sensorimotor Regions: Enhanced Motor Preparation and Control

The precuneus plays a crucial role in visuospatial processing and motor imagery [6,7,8]. The increased precuneus activity we observed in the HiP group specifically links this region to stepping accuracy. Accurate stepping requires integrating visual information with body-centered coordinates to form a spatial map, suggesting the precuneus significantly contributes to this process.

Increased activity in the postcentral gyrus suggests that sensory inputs, particularly plantar sensations, are processed with higher precision in individuals with superior stepping accuracy. This finding aligns with those of studies by Koenraadt et al. [49] and Haefeli et al. [50], who reported increased cortical activity during precision stepping and obstacle avoidance; however, our study specifically identifies the relevant frequency bands and anatomical regions involved.

The HiP group showed suppression in mu- and beta-band activity in the bilateral paracentral lobules, consistent with findings by Wagner et al. [25,51,52] and Seeber et al. [53,54]. This reduction in mu- and beta-band activity represents a well-established neurophysiological marker of motor planning and execution [55]. The event-related desynchronization (ERD) observed in the mu and beta bands from motor preparation through execution reflects fundamental mechanisms of motor control [25,26]. Research on voluntary gait control using BCI has reported sustained alpha/mu-band ERD associated with intentional control [56], supporting our findings in precision stepping.

Beta-band activity plays a critical role in motor and cognitive control [25,57], suggesting that the suppression pattern observed in the HiP group reflects smoother lower-limb motor control. A recent study by Nordin et al. [55] demonstrated that at faster gait speeds, the sensorimotor cortex shows reduced alpha and beta EEG spectral power, aligning with our observations that reduced mu- and beta-band activity is associated with skilled motor performance, suggesting a common neural mechanism for both stepping accuracy and gait speed modulation.

### 4.3. Visual Processing in the Occipital Region: Enhanced Visuospatial Integration

We observed changes in frequency band activity in the occipital lobes (middle occipital gyrus and cuneus). These regions are crucial for both initial visual processing and the integration of higher-order visuospatial information [58,59], essential for accurate foot placement.

The stepping task required participants to visually identify a target point and precisely align their heel with it, demanding efficient visual processing to create an accurate spatial map of the target location relative to the body. The distinct patterns of occipital activity in the HiP group suggest more effective visual information processing and integration with motor planning areas.

Bradford et al. [60] demonstrated that electrocortical activity can distinguish between different walking conditions. Our findings advance this understanding by showing that occipital activity patterns differentiate between high and low stepping accuracy, suggesting that precise stepping relies on optimized visual processing to inform motor planning.

Wagner et al. [51] showed that walking down a virtual alley activates premotor and parietal areas, emphasizing visuospatial processing in locomotion. Our results demonstrate that occipital processing efficiency directly relates to stepping performance, highlighting the importance of visual processing in precise foot placement. This visual–motor connection is further supported by studies showing electrocortical correlates of gait adaptation to visual kinematic perturbations [56], suggesting a critical visual–motor integration pathway for precision locomotion.

### 4.4. A Proposed Neural Network Framework for Stepping Accuracy

Integrating our findings, we propose a comprehensive neural network framework for stepping accuracy that builds upon current models of motor control. This proposed framework comprises the following three interconnected neural systems working in concert:

1. Error Monitoring System: Centered on the ACC, this system generates theta oscillations that monitor performance in real-time, detecting discrepancies between intended and actual foot placement. The enhanced theta activity in the HiP group suggests more efficient error monitoring processes facilitating precise stepping, aligning with findings from Luu et al. [27] and Jacobsen and Ferris [23] on ACC theta activity in error monitoring and locomotor adaptation.
2. Sensorimotor Integration System: Encompassing the paracentral lobules and postcentral gyrus, this system features task-specific mu and beta desynchronization patterns supporting flexible motor execution. The greater suppression of these rhythms in the HiP group may enable more precise motor adjustments, corresponding with findings from Wagner et al. [25] and Nordin et al. [55] on active locomotion and adaptation.
3. Visuospatial Processing System: Including the precuneus and occipital regions, this system processes visual information and integrates it with body-centered coordinates to create a spatial map for guiding foot placement. Enhanced activity in these regions among high performers suggests more effective visuospatial processing, consistent with studies by Wagner et al. [51] on parietal regions in locomotion.

We hypothesize that these systems interact dynamically throughout stepping execution. The error monitoring system likely modulates the sensorimotor integration system through top-down control, while the visuospatial processing system provides essential spatial information guiding motor planning and execution. Enhanced coordination between these systems appears associated with superior stepping accuracy; however, further research is needed to validate this proposed framework and examine the causal relationships between these systems.

This integrated framework extends traditional motor control models by emphasizing the role of cognitive monitoring processes in precise motor execution. Rather than viewing motor control as primarily bottom-up, our findings suggest the importance of top-down cognitive control mechanisms in fine-tuning motor output. This perspective is supported by electrocortical evidence from BCI-controlled walking studies [27] and split-belt adaptation paradigms [23].

### 4.5. Study Limitations

Some limitations should be considered when interpreting our results. Our study included only young male participants, limiting generalizability to females, older adults, and individuals with neurological disorders. Sex differences in neural control mechanisms have been documented [22,23], underscoring the importance of examining whether the neural correlates of stepping accuracy are consistent across sexes. While we focused on the initial step movement to isolate neural mechanisms of step accuracy, future studies should examine how these mechanisms function during more complex walking tasks involving obstacle avoidance or dual-task conditions. The laboratory environment may not fully capture the neural dynamics of stepping in real-world environments with varying surfaces and distractions. Our sample size, while sufficient to detect significant differences between groups, could be expanded in future studies to enhance statistical power. 

Future research should investigate these neural mechanisms in clinical populations with gait impairments. The frequency-specific oscillatory patterns identified could serve as potential biomarkers for fall risk assessment and as targets for neuromodulation or neurofeedback interventions aimed at enhancing stepping accuracy in vulnerable populations.

## 5. Conclusions

Our study provides novel insights into the neural oscillatory mechanisms underlying stepping accuracy. We demonstrated that accurate step execution relies on a distributed yet specialized neural network comprising (1) enhanced performance monitoring via ACC theta-band activity; (2) effective sensorimotor integration through mu- and beta-band suppression in motor areas; and (3) efficient visuospatial processing in parietal and occipital regions.

The frequency-specific oscillatory patterns identified have important clinical implications as potential biomarkers for assessing fall risk and as targets for interventions to improve stepping accuracy. Our methodological approach using eLORETA-ICA effectively addressed common challenges in mobile EEG research by separating neural signals from movement-related artifacts. By elucidating these neural mechanisms, our findings provide a foundation for developing more effective rehabilitation strategies and fall prevention programs targeting the specific neural systems involved in precise foot placement.

## Figures and Tables

**Table 1:** Participant characteristics (*n* = 16).

Characteristic	Mean ± SD or *n* (%)
Age (years)	23.2 ± 2.7
Height (cm)	169.9 ± 4.5
Weight (kg)	62.4 ± 6.9
Dominant foot (Right/Left)	13 (81.3)/3 (18.7)
Sleep duration, previous night (hours)	6.2 ± 1.4
MMSE (points)	30 (100)
MMSE: Mini-Mental State Examination; SD: standard deviation.	
All participants scored 30 points on the MMSE, indicating no cognitive impairment.	

**Table 2:** Areas of neural activity in the seven clusters.

	HiP, *n* = 8			LoP, *n* = 8		
Cluster	Brain Lobe	Region	BA	Brain Lobe	Region	BA
1	Right parietal lobe	Postcentral gyrus	3	No EEG activity		
2	Left frontal lobe	Superior frontal gyrus	8	Bilateral parietal lobes	Superior parietal lobule	7
3	Right parietal lobe	Precuneus	7	No EEG activity		
4	Left occipital lobe	Middle occipital gyrus	19	Bilateral frontal lobes	Superior frontal gyrus	8
5	Limbic lobe	Anterior cingulate cortex	32	No EEG activity		
6	No EEG activity			Bilateral parietal lobes	Paracentral lobule	4
7	No EEG activity			Left occipital lobe	Cuneus	19
HiP: high-performing group characterized by a small error distance between the target point and the heel contact point; LoP: low-performing group characterized by a large error distance between the target point and the heel contact point; BA: Brodmann area; EEG: electroencephalography.						

**Table 3:** The microstate probabilities for each frequency band in the seven clusters.

Cluster	Total	δ	θ	α (μ)	β
1	7.69	7.69	7.69	7.69	7.69
2	15.39	15.39	15.39	15.39	15.39
3	17.31	23.08	15.39	15.39	15.39
4	5.77	0.00	7.69	7.69	7.69
5	21.15	23.08	38.46	15.39	7.69
6	17.31	15.39	7.69	23.08	23.08
7	15.39	15.39	7.69	15.39	23.08

δ: delta; θ: theta; α: alpha; β: beta. Each value represents a percentage.

**Figure A1:**
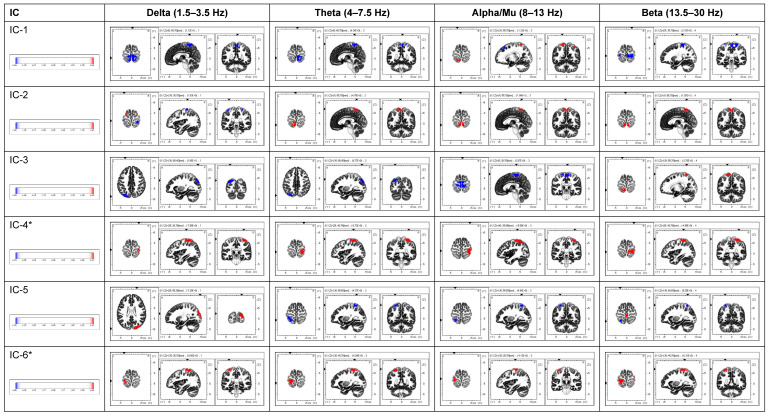

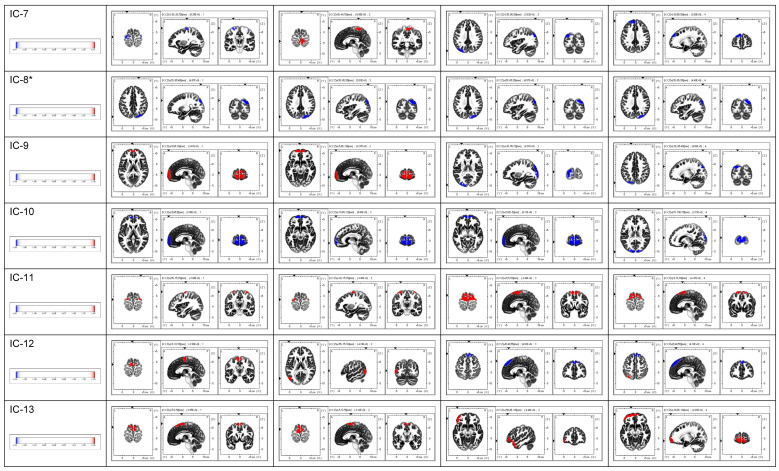
Images of 13 independent components (ICs) within their respective frequency bands obtained using eLORETA-ICA on EEG data during the stepping task. Each row represents a different IC, and columns represent the four frequency bands analyzed: Delta (1.5–3.5 Hz), Theta (4–7.5 Hz), Alpha/Mu (8–13 Hz), and Beta (13.5–30 Hz). Ten ICs (IC-1, IC-2, IC-3, IC-5, IC-7, IC-9, IC-10, IC-11, IC-12, and IC-13) were identified as physiological neural network activity, while three ICs (IC-4, IC-6, and IC-8) were classified as artifacts based on their spatial and spectral characteristics. In the color-coded maps, red represents increased power, whereas blue indicates decreased power as IC activity increases. * Note: IC-4, IC-6, and IC-8 were identified as artifacts and excluded from subsequent analyses.

## Data Availability

Some of the participant data presented in this study are available upon request from the corresponding author. However, due to privacy concerns, certain participants’ data may not be offered.

## Abbreviations

The following abbreviations are used in this manuscript:

ACC	Anterior cingulate cortex
BCI	Brain–computer interface
ED	Error distance
EEG	Electroencephalography
eLORETA	Exact low-resolution electromagnetic tomography
EMG	Electromyography
EOG	Electrooculography
MMSE	Mini-mental state examination
ERD	Event-related desynchronization
fMRI	Functional magnetic resonance imaging
HiP group	High-performing group
ICA	Independent component analysis
ICs	Independent components
LoP group	Low-performing group
MoBI	Mobile brain/body imaging
MSA	Microstate segmentation analysis
PPC	Posterior parietal cortex
